# Phoenixin-20 Stimulates mRNAs Encoding Hypothalamo-Pituitary-Gonadal Hormones, is Pro-Vitellogenic, and Promotes Oocyte Maturation in Zebrafish

**DOI:** 10.1038/s41598-020-63226-x

**Published:** 2020-04-14

**Authors:** Jithine Jayakumar Rajeswari, Suraj Unniappan

**Affiliations:** Laboratory of Integrative Neuroendocrinology, Department of Veterinary Biomedical Sciences, Western College of Veterinary Medicine, University of Saskatchewan, S7N 5B4 Saskatchewan, Canada

**Keywords:** Animal physiology, Zoology, Physiology, Reproductive biology

## Abstract

Phoenixin-20 (PNX-20) is a bioactive peptide with hormone-like actions in vertebrates. In mammals, PNX stimulates hypothalamo-pituitary-gonadal hormones and regulate reproductive processes. Our immunohisto/cytochemical studies show PNX-like and the putative PNX receptor, SREB3-like immunoreactivity in the gonads of zebrafish, and in zebrafish liver (ZFL) cells. Intraperitoneal injection of zebrafish PNX-20 upregulates mRNAs encoding both salmon gonadotropin-releasing hormone (GnRH), and chicken GnRH-II and kisspeptin and its receptor in zebrafish hypothalamus. Similarly, luteinizing hormone receptor mRNA expression in the testis, follicle-stimulating hormone receptor in the ovary, and the kisspeptin system were upregulated in the gonads of PNX-20 injected fish. We also observed the upregulation of genes involved in the sex steroidogenic pathway (*cyp11a1*, *cyp17a1*, *17βhsd*, *cyp19a1a*) in the gonads of PNX-20 administered fish. PNX-20 upregulates the expression of vitellogenin isoforms and estrogen receptor (*esr2a* and *2b*) mRNAs in ZFL cells *in vitro*. Meanwhile, siRNA-mediated knockdown of PNX-20 resulted in the downregulation of all vitellogenin transcripts, further suggesting its possible role in vitellogenesis. PNX-20 treatment resulted in a significant increase in germinal vesicle breakdown in zebrafish follicles *in vitro*. Collectively, these results provide strong evidence for PNX-20 effects on the HPG axis and liver to promote reproduction in zebrafish.

## Introduction

Advances in computational biology, especially in the use of bioinformatics tools, helped in the discovery of many regulatory molecules with hormone-like actions. A recent example is phoenixin-20 (PNX-20), which was originally identified as a reproductive regulatory peptide^1^. Phoenixin was first isolated from the rat hypothalamus and bovine heart by two independent research groups^1,2^. The mature phoenixin peptide is produced from an uncharacterized protein called small integral membrane protein 20 (SMIM20) or chromosome 4 open reading frame 52 (C4orf52). Phoenixin is reported to be present in two active forms, a 20 amino acid peptide named PNX-20 and a 14 amino acid one called PNX-14^1,2^. PNX-14 is identical in humans, rats, mice, pigs, and dogs. PNX-20 differs in one amino acid between the coding regions of human, canine and porcine sequences^1^. It was reported that PNX is expressed in various parts of the brain and peripheral tissues in mammals, with the highest expression in the hypothalamus. Treen *et al*., reported that a putative receptor of PNX is the G protein-coupled receptor 173 (GPR173), which is also called super conserved receptor expressed in brain 3 (SREB3)^3^.

PNX stimulates reproductive functions via acting on the HPG axis^1^. Later it was reported that PNX mediates its reproductive regulatory effects through GPR173, and cAMP-PKA dependent pathway, acting on both gonadotropin-releasing hormone (GnRH) and kisspeptin 1 (Kiss1) expressing neurons^3^. In mice, PNX positively influences Kiss1 transcription and GnRH mediated gonadotropin release^1,4^. Intracerebroventricular (ICV) injection of PNX-20 significantly increases plasma luteinizing hormone (LH) levels in a dose-related manner, and potentiate GnRH-induced LH secretion from cultured anterior pituitary cells *in vitro*^5^. Although PNX is originally identified and described as a regulator of reproductive functions, non-reproductive roles of PNX are also reported later on. The non-reproductive functions of PNX include the regulation of pain sensation^2^, memory retention^6^, and food intake^7,8^, cardiac functions^9^, vasopressin release^10^, and the proliferation and differentiation of white adipose (3T3-L1) cells^11^. Overall, PNX is now known to have multiple biological effects.

PNX in non-mammals is poorly understood. In fish (spotted scat; *Scatophagus argus*), short term fasting increases the expression of PNX-20 in the hypothalamus and refeeding attenuates it^8^. It was also reported that both *in vitro* and *in vivo* administration of PNX upregulates the expression of *gnrh* receptor (*gnrhr*), *lh* and *fsh* in the pituitary of spotted scat^12^. These results suggest PNX appears to have a regulatory role in reproduction in fish. This research aimed to determine whether PNX-20 has a reproductive regulatory role in zebrafish, a well-characterized model system in comparative endocrinology. We tested for possible effects of PNX-20 on the tissue-specific expression of mRNAs encoding reproductive regulatory peptides, and vitellogenin genes in the liver, and oocyte maturation in zebrafish. Our results indicate that PNX-20 is a positive modulator of the HPG axis and vitellogenesis, and promotes oocyte maturation in zebrafish.

## Results

### PNX-like and SERB3-like immunoreactivity (ir) was detected in the gonads of zebrafish

**Figure 1:**
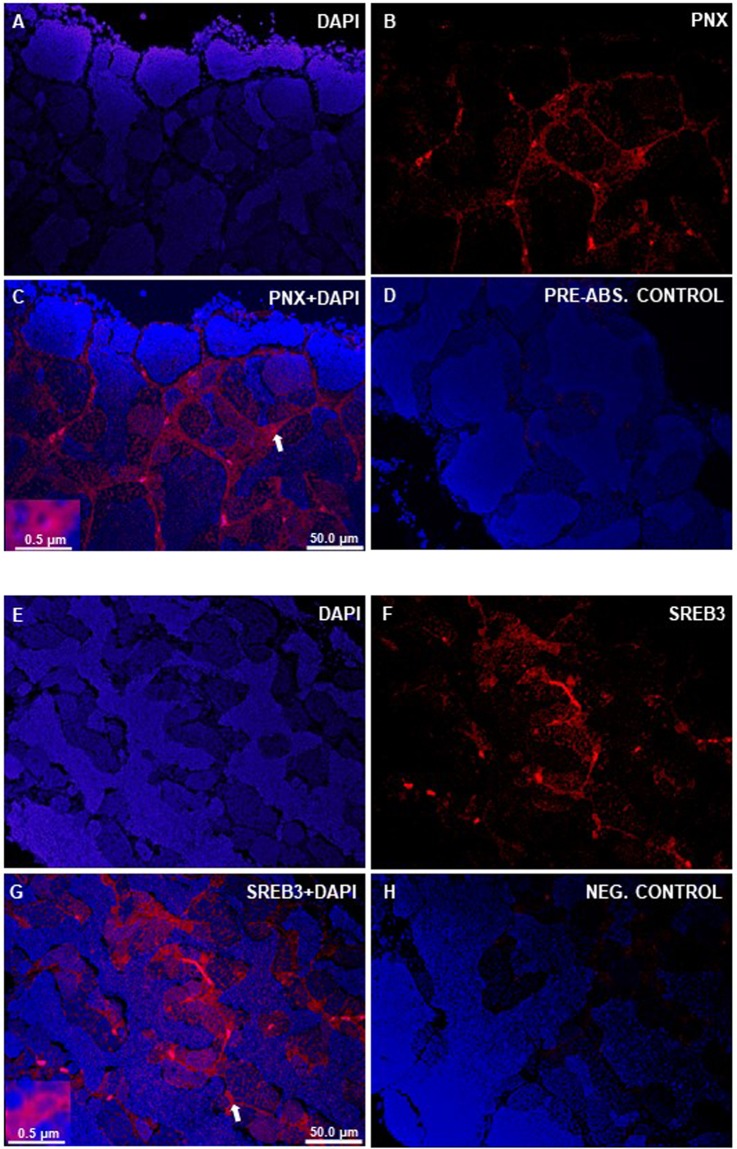
Immunohistochemical localization of PNX-like and SREB3-like- ir in the testis of zebrafish. Figure shows representative sections of zebrafish testis (**A–H**) showing PNX-like (red; **B,C**) and SREB3-like (red; **F,G**) immunoreactivity. No PNX/SREB-3-like-ir was observed in both pre-absorption (**D**) and secondary antibody alone negative control (**H**). Nuclei shown in blue are stained with DAPI. Scale bars are indicated in each image.

**Figure 2:**
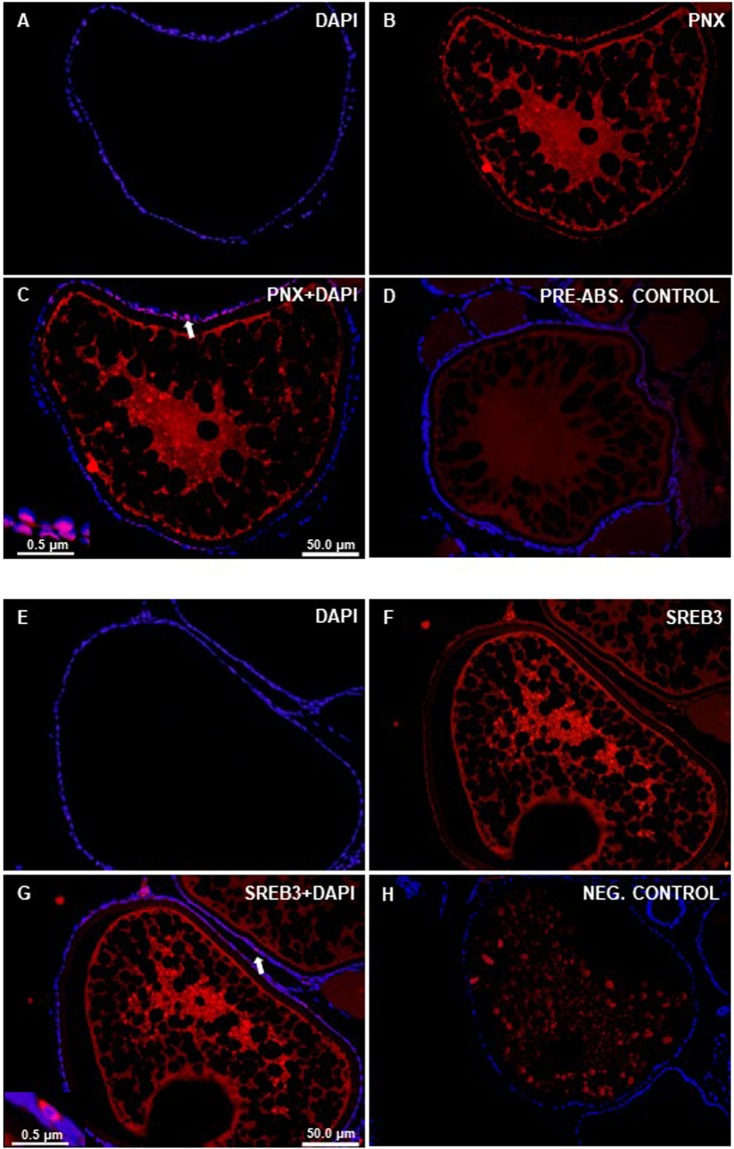
Immunohistochemical localization of PNX-like and SREB3-like- ir in the ovary of zebrafish. Figure shows representative sections of zebrafish ovary (**A–H**) showing PNX-like (red; **B,C**) and SREB3-like (red; **F,G**) immunoreactivity. No PNX/ SREB-3-like-ir was observed in both pre-absorption (**D**) and secondary antibody alone-negative controls (**H**). Nuclei shown in blue are stained with DAPI. Scale bars are indicated in each image.

In the gonads of both male (Fig. 1) and female (Fig. 2) zebrafish, we found PNX-like and SREB3-like immunoreactivity in the supporting cell layers. No immunoreactive signals were found in the pre-absorption or secondary antibody alone negative controls for testis and ovary.

### PNX-like and SERB3-like immunoreactivity (ir) was detected in the zebrafish liver cell (ZFL) line

**Figure 3:**
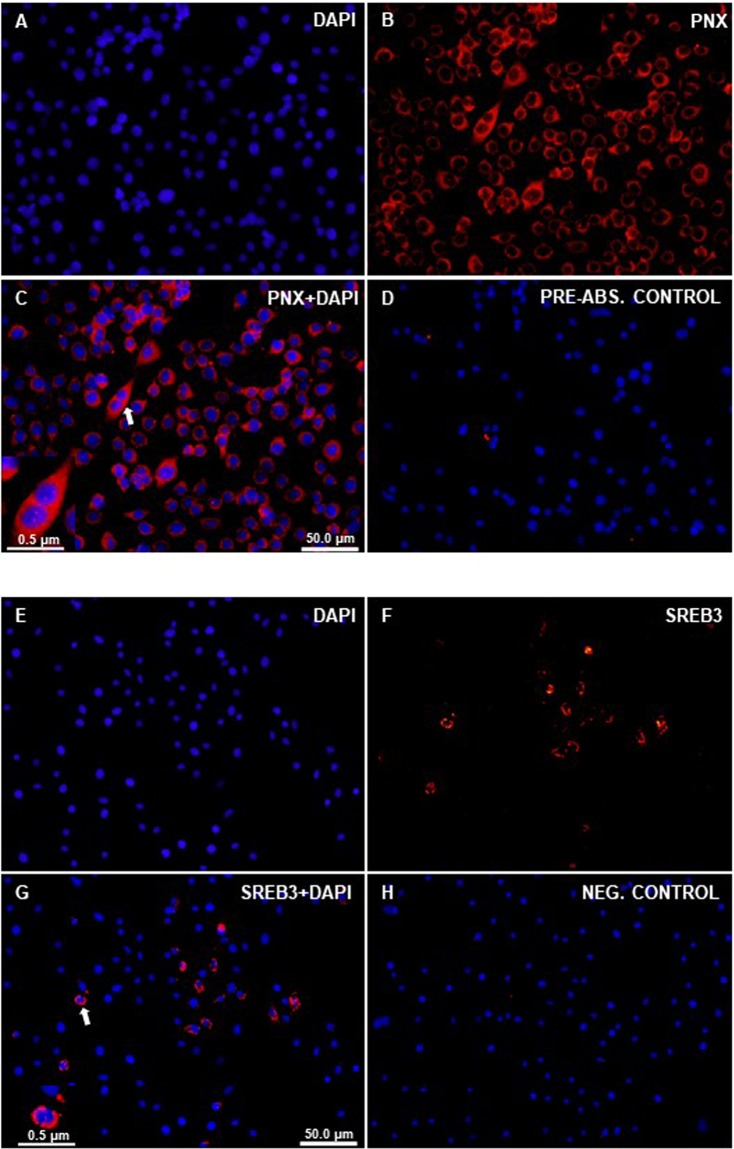
Immunohistochemical localization of PNX-like and SREB3-like- ir in the zebrafish liver cell line. Figure shows representative sections of zebrafish liver cell line (**A–H**) showing PNX-like (red; **B,C**) and SREB3-like (red; **F,G**) immunoreactivity. No PNX/SREB-3-like-ir was observed in both pre-absorption (**D**) and secondary antibody alone-negative controls (**H**). Nuclei are shown in blue are stained with DAPI. Scale bars are indicated in each image.

Both PNX-like and SREB3-like immunoreactivity was detected in the cytoplasm of ZFL cells (Fig. 3). No immunostaining was found in pre-absorption and negative controls.

### Intraperitoneally administered PNX-20 upregulates GnRH, kisspeptin and brain aromatase mRNA expression in the hypothalamus of zebrafish

**Figure 4:**
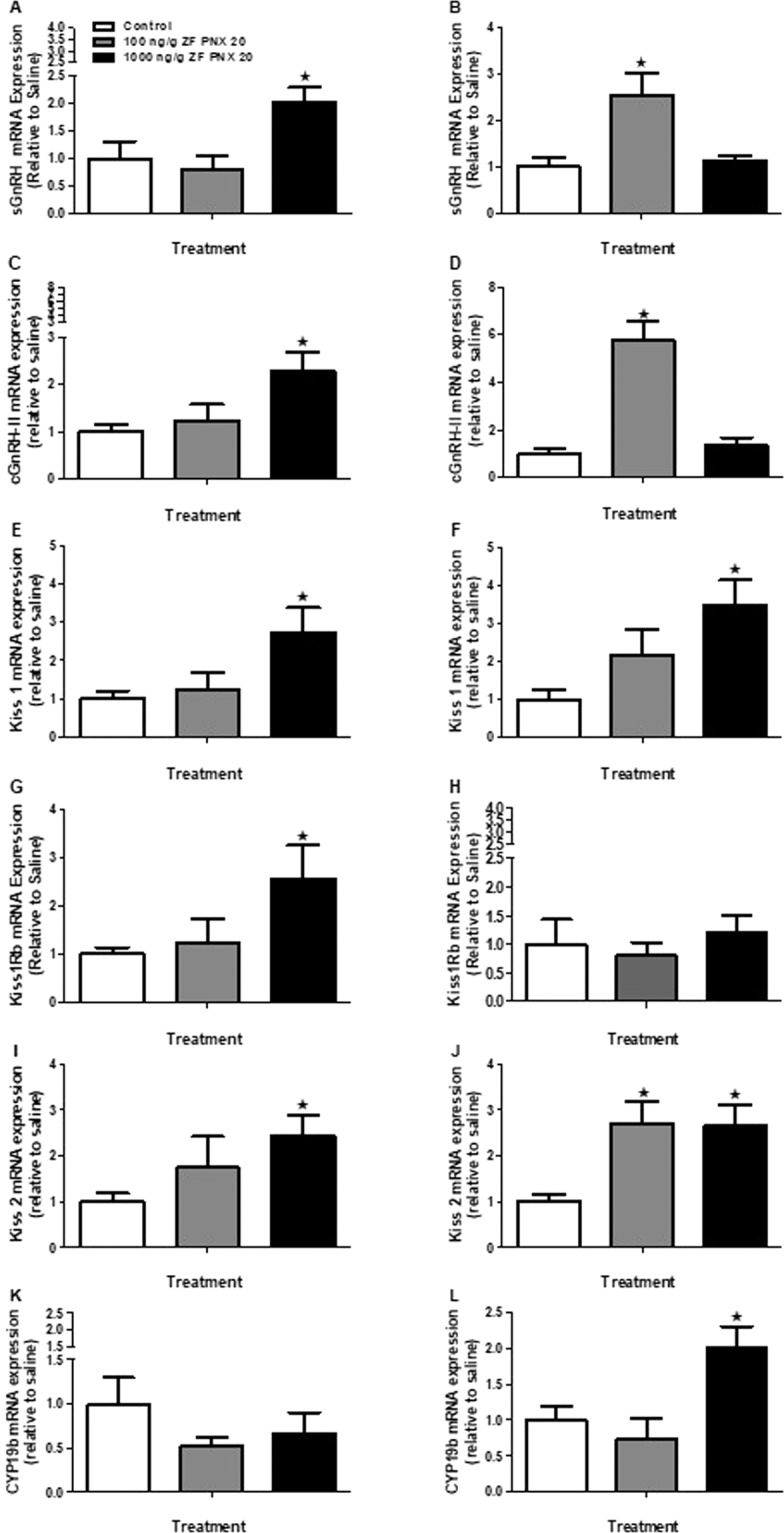
Expression levels of mRNAs encoding reproductive regulatory genes in the hypothalamus (A-L) of male (left panel) and female (right panel) zebrafish 1 h after intraperitoneal administration of 100 and 1000 ng/g bw of PNX-20. PNX administration significantly upregulates the mRNA expression of *sgnrh* (**A**,**B**), *cgnrh-II* (**C,D**), kiss-1 (**E**,**F**) and *kiss-*2 (**I**,**J**) in both male and female fish group. The *kiss1rb* (**G**) significantly upregulated in the male treatment group only. The brain aromatase (*cyp19a1b*) significantly upregulated in the female treatment group only (**L**). Data obtained by RT-qPCR are represented as mean + SEM (n = 6 fish/group). One-way ANOVA followed by Tukey’s multiple comparison test or Student–Newman–Keuls (SNK) test was used for statistical analysis. Asterisks denote significant differences between control and treated groups. p < 0.05 was considered statistical significance.

A significant increase in salmon-type gonadotropin-releasing hormone (*sgnrh* or *gnrh3*) and chicken gonadotropin-releasing hormone (*cgnrh-II* or *gnrh2*) mRNA expression was noted in the hypothalamus of both male and female zebrafish post-PNX-20 administration (Fig. 4A–D). We observed a significant increase in the expression of kisspeptin 1 (*kiss1*, in both male and female treatment groups) (Fig. 4E,F**)** and its receptor (*kiss1rb*; only in the male fish group) (Fig. 4G) mRNAs in zebrafish hypothalamus. However, we did not observe any significant changes in the mRNA expression of *kiss1rb* in the hypothalamus of female fish (Fig. 4H). We also found a significant increase in the expression of kisspeptin 2 (*kiss 2*) mRNA in the hypothalamus of both male (1000 ng/g PNX-20) and female (both 100 and 1000 ng/g PNX-20) treatment groups (Fig. 4I,J). The brain aromatase (cytochrome P450, family 19, subfamily A, polypeptide 1b, *cyp19a1b*) mRNA expression was also significantly increased in the hypothalamus of female fish injected with 1000 ng/g PNX-20 (Fig. 4L), but no significant changes were found in the male fish group treated with PNX-20 (Fig. 4K).

### PNX-20 influence the expression of reproductive regulatory genes in the gonads of zebrafish

**Figure 5:**
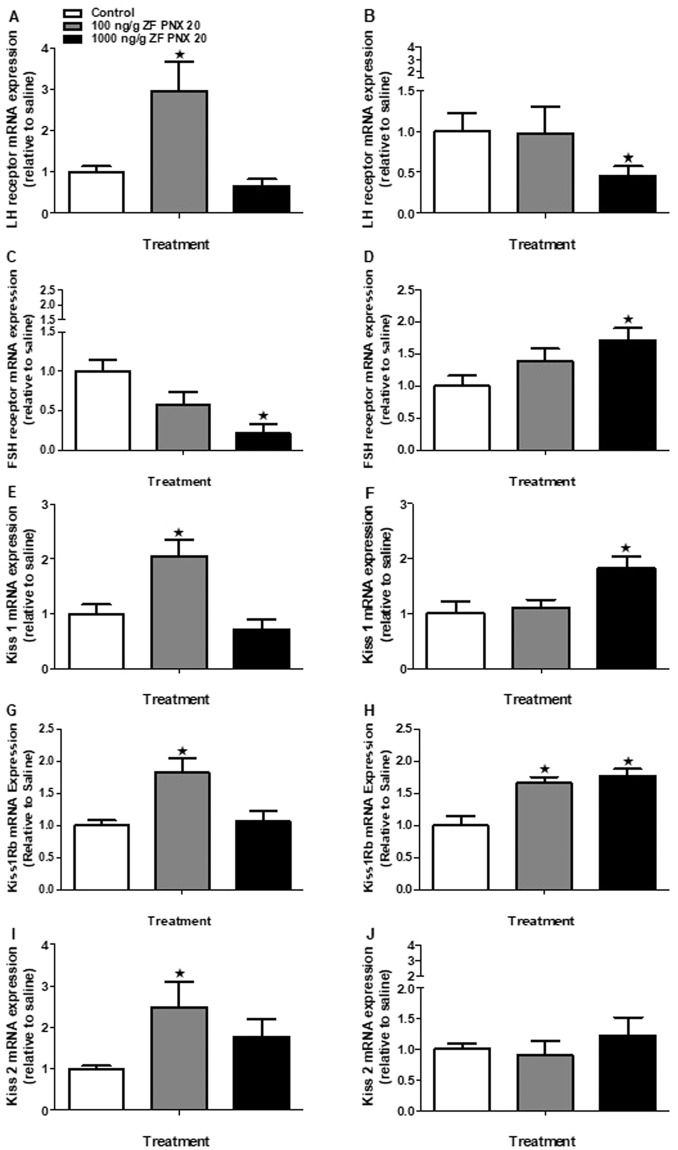
Expression levels of mRNAs encoding gonadotropin receptors and kisspeptin system in the gonads (A-J) of male (left panel) and female (right panel) zebrafish post-PNX-20 administration. Upregulation of the mRNA expression of *lhr* in the testis (**A**) and downregulation in the ovary (**B**) of zebrafish was observed post-PNX-20 injection. However, *fhsr* mRNA expression downregulated in the testis (**C**) and upregulated in the ovary (**D**) post-PNX administration. The *kiss-1* (**E,F**) and *kiss1rb* (**G,H**) mRNA expression significantly upregulated in both sexes. Significant increase in the kiss-2 mRNA expression observed only in the testis of the treatment group (**I**). Data obtained by RT-qPCR are represented as mean + SEM (n = 6 fish/group). One-way ANOVA followed by Tukey’s multiple comparison test or Student–Newman–Keuls (SNK) test was used for statistical analysis. Asterisks denote significant differences between control and treated groups. p < 0.05 was considered statistical significance.

Sex-specific expression of *lh* receptor (*lhr*) and *fsh* receptor (*fshr*) mRNAs was observed in the gonads of zebrafish post-PNX-20 administration. *lhr* mRNA expression was significantly increased in the testis (100 ng/g PNX-20), and significantly decreased (1000 ng/g PNX-20) in the ovary of zebrafish (Fig. 5A,B). The *fshr* mRNA expression was significantly downregulated in the testis and significantly upregulated in the ovary of zebrafish injected with 1000 ng/g PNX-20 (Fig. 5C,D). A significant increase in *kiss1* and *kiss1rb* mRNA expression in the testis and ovary of zebrafish treatment groups (Fig. 5E–H), and *kiss*2 mRNA expression in the testis of zebrafish injected with 100 ng/g PNX-20 (Fig. 5I) was detected. However, we didn’t observe any significant changes in the expression of *kiss2* mRNA expression in the ovary of PNX 20 treated fish (Fig. 5J).

### PNX-20 influences genes involved in the gonadal sex steroidogenic pathway in zebrafish

**Figure 6:**
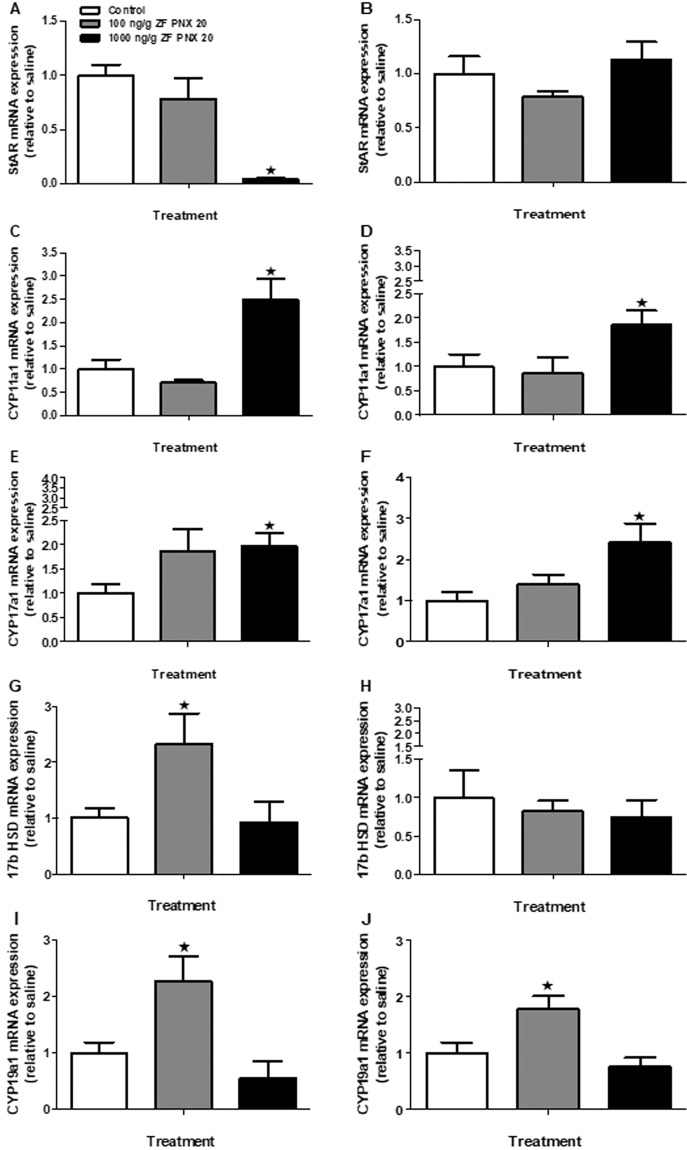
Expression pattern of genes involved in the sex steroidogenic pathway in the gonads (A-J) of male (left panel) and female (right panel) zebrafish post-PNX-20 administration. A significant decrease in *star mRNA* expression was observed in the testis post-PNX-20 injection (**A**). PNX-20 upregulates the mRNA expression of *cyp11a1* (**C,D**), *cyp17a1* (**E,F**), *cyp19a1a* (**I,J**) in the gonads of male and female zebrafish. The *17βhsd* upregulated only among the testis of treatment groups (**G**). Data obtained by RT-qPCR are represented as mean + SEM (n = 6 fish/group). One-way ANOVA followed by Tukey’s multiple comparison test or Student–Newman–Keuls (SNK) test was used for statistical analysis. Asterisks denote significant differences between control and treated groups. p < 0.05 was considered statistical significance.

A significant decrease in steroidogenic acute regulatory protein (*star*) mRNA expression in the testis of 1000 ng/g PNX-20 treated fish group (Fig. 6A) was found. However, no significant changes in *star* mRNA expression were observed in the ovary of PNX treated fish (Fig. 6B). Cytochrome P450 family 11 subfamily A member 1 (*cyp11a1*, or the cholesterol side-chain cleavage enzyme) (Fig. 6C,D) and the cytochrome P450 family 17 subfamily A member 1 (*cyp17a1*, cytochrome P450 monooxygenase) (Fig. 6E,F) mRNA expression were significantly elevated in the gonads post-PNX-20 (1000 ng/g) administration. A significant increase in the expression of 17-beta hydroxysteroid dehydrogenase (*17βhsd*) was observed in the testis of zebrafish treated with 100 ng/g PNX-20 (Fig. 6G), but no significant changes were observed in the female fish (Fig. 6H). Similarly, the expression of cytochrome P450 family 19 subfamily A member 1 (*cyp19a1a*, gonadal aromatase) mRNA in the gonads of both male and female zebrafish was upregulated post 100 ng/g PNX-20 (Fig. 6I,J).

**Figure 7:**
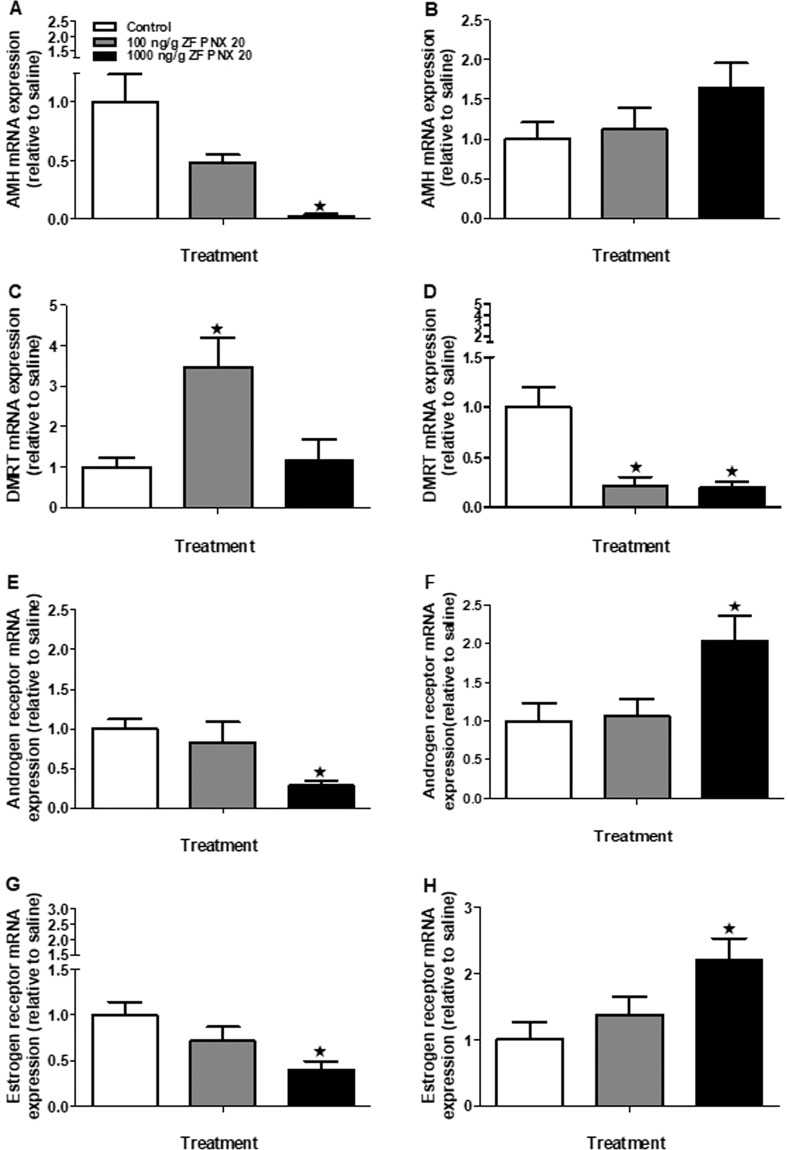
mRNA expression pattern of *amh*, *dmrt1* and sex-steroid hormone receptors in the gonads (A-H) of male (left panel) and female (right panel) zebrafish post-PNX-20 administration. The *amh* mRNA expression significantly decreased in the testis (**A**) of PNX injected fish group. However, *dmrt1 mRNA* significantly increased in the testis (**C**) and significantly decreased in the ovary (**D**). Both *ar* and *er* mRNAs downregulated in the testis (**E**,**G**). In the ovary of PNX injected fish *ar* and *er* mRNA’s upregulated (**F**,**H**). Data obtained by RT-qPCR are represented as mean + SEM (n = 6 fish/group). One-way ANOVA followed by Tukey’s multiple comparison test or Student–Newman–Keuls (SNK) test was used for statistical analysis. Asterisks denote significant differences between control and treated groups. p < 0.05 was considered statistical significance.

Among gonadal genes that are important in zebrafish reproduction, anti-Müllerian hormone (*amh*) was significantly downregulated in the testis of zebrafish injected with 1000 ng/g PNX-20 (Fig. 7A). However, no significant changes in *amh* mRNA expression was observed in female fish injected with PNX-20 (Fig. 7B). Meanwhile, *dmrt1* mRNA in the testis (100 ng/g) was increased (Fig. 7C) but was decreased (100 & 1000 ng/g) in the ovary (Fig. 7D). In the testis of 1000 ng/g PNX-20 treatment groups, both androgen receptor (*ar*) and estrogen receptor (*er*) mRNA expression was significantly downregulated (Fig. 7E,G). However, in the ovary of 1000 ng/g PNX-20 treated fish, both *ar* and *er* mRNA expression was significantly increased (Fig. 7F,H).

### *In vivo* treatment of PNX-20 upregulates the expression of vitellogenin system mRNAs in ZFL cells

**Figure 8:**
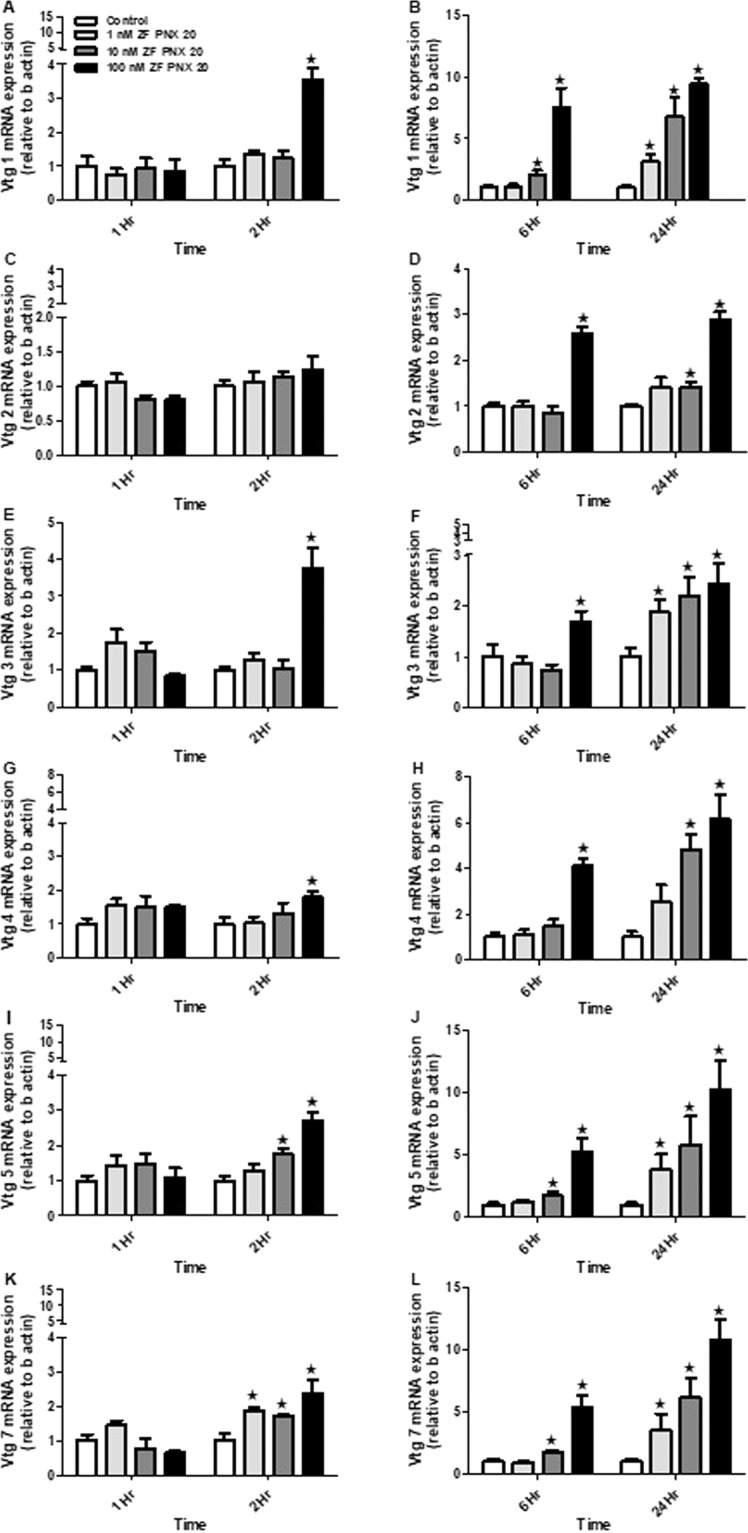
Vitellogenin mRNA expression pattern in the ZFL cells post-PNX-20 treatment (A-L). The left panel represents 1 and 2 hr and the right panel represents 6 and 24 hr treatment groups. No significant changes in any of the vitellogenin mRNA expression was observed in 1 hr treatment groups (1hr- **A**,**C**,**E**,**G**,**I**,**K**). Among 2 hr treatment groups, *vtg 1*, *vtg 3*, *vtg 4*, *vtg 5* and *vtg 7* mRNA expression was upregulated (2 hr- **A**,**E**,**G**,**I**,**K**). However, significant upregulation of all *vtg’s* was observed in the 6 and 24 hr treatment groups (**B**,**D**,**F**,**H**,**J**,**L**). Graphs represent pooled data from 2 independent study (‘n’ = 6 wells/treatment). One-way ANOVA followed by Tukey’s multiple comparison test or Student–Newman–Keuls (SNK) test was used for statistical analysis. Asterisks denote significant differences between control and treated groups. p < 0.05 was considered statistical significance.

A significant increase in the *vtg 1* mRNA expression was detected at 2, 6 and 24 hr post-PNX-20 treatment (Fig. 8A,B). As for *vtg 2*, we observed a significant increase in the expression at 6 and 24 hr post-PNX-20 treatment groups only (Fig. 8D), but not at 1 and 2 hr after PNX-20 treatment (Fig. 8C). However, similar to what we observed in case of *vtg 1*, the mRNA expression of *vtg 3*, 4, 5 and 7 were significantly upregulated at 2, 6 and 24 hr post-PNX-20 treatment (Fig. 8E–L).

### PNX-20 upregulates the mRNA expression of estrogen receptor 2 (*esr2*) and sex hormone-binding globulin (*shbg*) in ZFL cells

**Figure 9:**
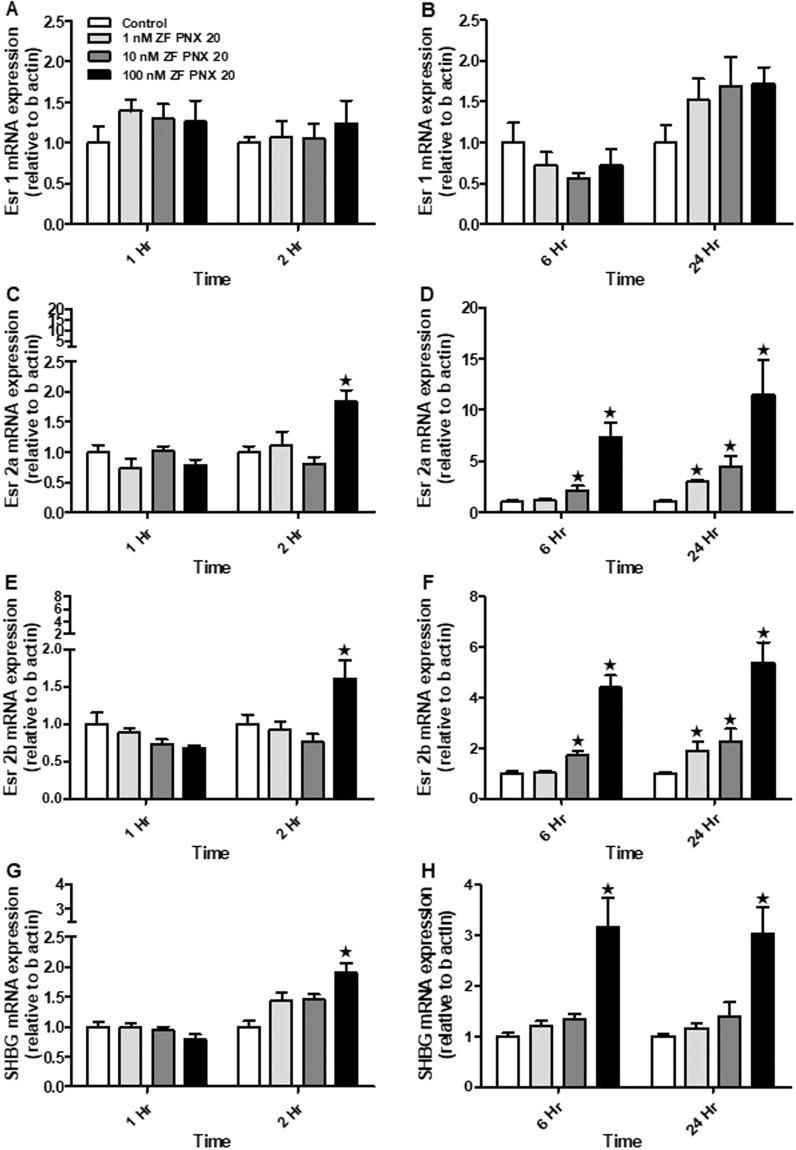
Expression pattern of estrogen receptor and *shbg* mRNA post-PNX-20 incubation in ZFL cells. The left panel represents 1 and 2 hr and the right panel represents 6 and 24 hr treatment groups. The mRNA expression of *esr1a* was not changed post-PNX- 20 treatment under any of the time points considered (**A**,**B**). Both *esr2a*, *esr2b* and *shbg* mRNA expression upregulated (**C**–**H**) in 2, 6 and 24 hr PNX-20 treatment groups. Graphs represent pooled data from 2 independent study (‘n’ = 6 wells/treatment). One-way ANOVA followed by Tukey’s multiple comparison test or Student–Newman–Keuls (SNK) test was used for statistical analysis. Asterisks denote significant differences between control and treated groups. p < 0.05 was considered statistical significance.

PNX-20 treated caused a significant increase in *esr-2a* and *esr-2b* mRNA expression (Fig. 9C–F). However, we didn’t observe any significant changes in the expression of *esr-*1 mRNA expression in any treatment group (Fig. 9A,B). PNX-20 upregulated *shbg* mRNA expression at 2, 6 and 24 h (Fig. 9G,H).

### Significant reduction in *smim*2*0* mRNA expression was observed in the ZFL cells post siRNA treatments

**Figure 10:**
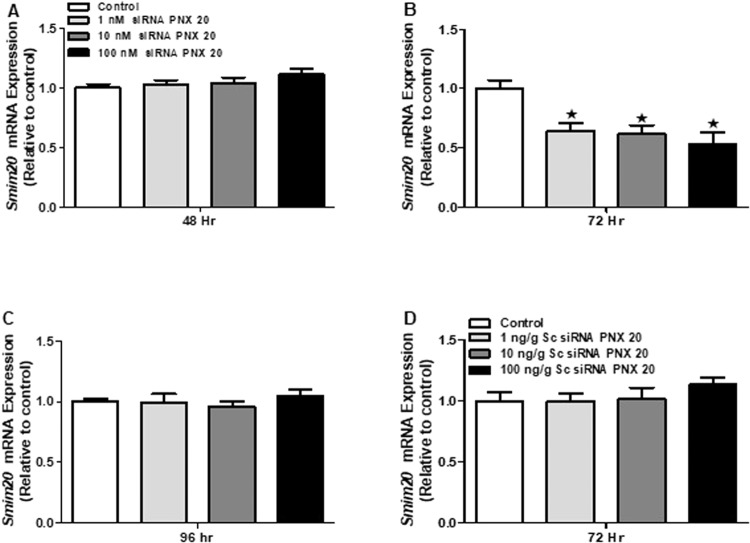
mRNA expression of *smim20* in the ZFL cells post siRNA and scrambled siRNA treatment. No significant changes in *smim20* mRNA expression was observed in siRNA treated cells at 48 and 96 hr time points (**A**,**C**). A significant decrease in *smim20* mRNA expression was observed at 72 hr post siRNA treatment (**B**) in all concentrations we tested compared to control. No significant changes in the mRNA expression of *smim20* was observed among the scrambled siRNA treated groups (**D**). Graphs represent pooled data from 2 independent study (‘n’ = 6 wells/treatment). One-way ANOVA followed by Tukey’s multiple comparison test or Student–Newman–Keuls (SNK) test was used for statistical analysis. Asterisks denote significant differences between control and treated groups. p < 0.05 was considered statistical significance.

The siRNA mediated knockdown resulted in a significant reduction in *smim20* mRNA in ZFL cell line 72 hr post-incubation (Fig. 10B). However, no significant changes in *smim20* mRNA expression was observed among 24 (data not included), 48 and 96 hr scrambled siRNA treatment groups (Fig. 10A,C). In addition, we didn’t observe any significant changes in *smim20* mRNA expression in scrambled siRNA group post-72 hr treatment (Fig. 10D).

### siRNA mediated PNX-20 knockdown downregulates *vtg* mRNAs in ZFL cells

**Figure 11:**
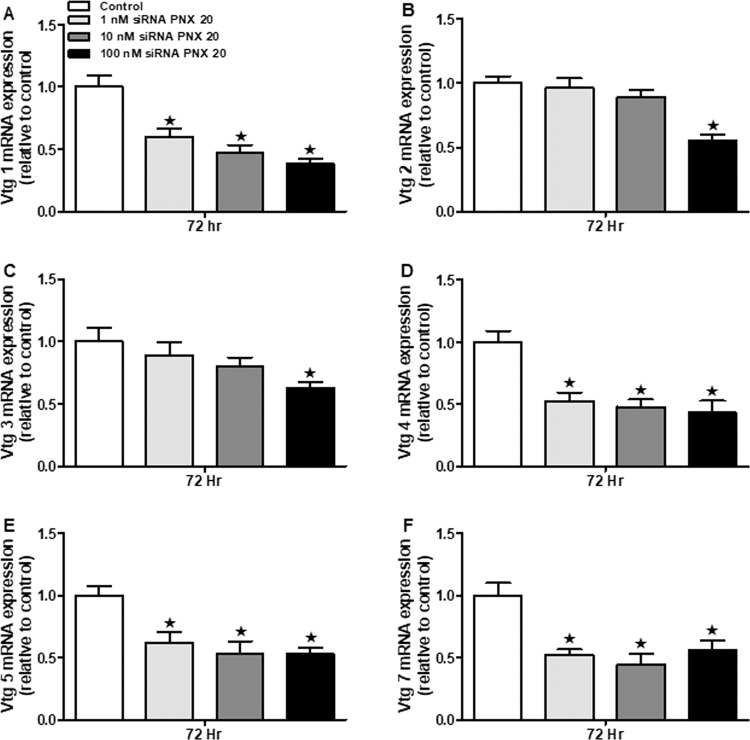
Effect of siRNA mediated gene knockdown of *smim20* in vitellogenin mRNA expression profile in ZFL cells. Reduction in the mRNA expression of *smim20* downregulates the expression of all *vtg* mRNA’s in the ZFL cells (**A–F**). Graphs represent pooled data from 2 independent study (‘n’ = 6 wells/treatment). One-way ANOVA followed by Tukey’s multiple comparison test or Student–Newman–Keuls (SNK) test was used for statistical analysis. Asterisks denote significant differences between control and treated groups. p < 0.05 was considered statistical significance.

All *vtg* (*vtg 1*, *2*, 3, 4, 5 & 7) mRNAs were significantly downregulated in the siRNA treated group (Fig. 11A–F). We found no significant change in the expression of any of the *vtg* mRNAs in cells treated with the scrambled siRNA (data not shown).

### PNX-20 increased germinal vesicle breakdown and oocyte maturation in zebrafish

**Figure 12:**
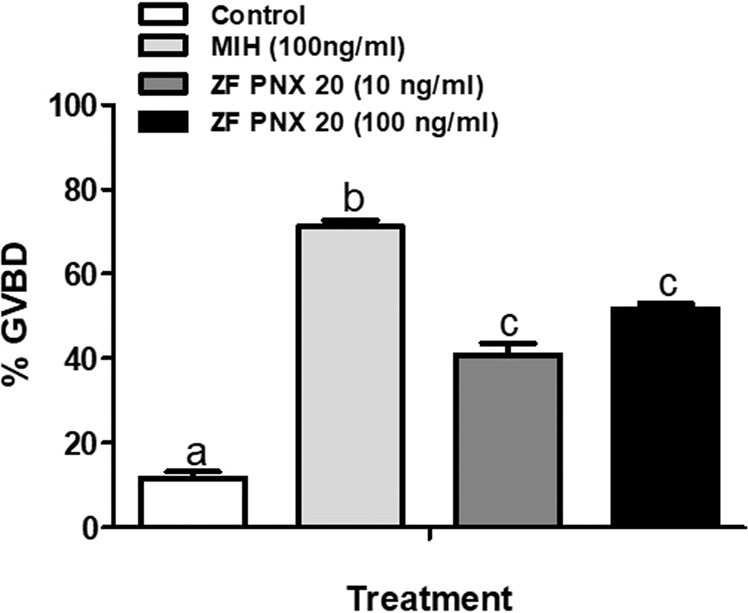
PNX-20 promotes germinal vesicle breakdown and oocyte maturation in zebrafish. A significant increase in the maturation of oocytes were observed in ovarian follicles incubated with PNX-20 (**A**) compared to controls. However, follicles treated with MIH showed highest maturation rates compared to control and PNX treated group. Graphs represent pooled data from four independent study (n’ = 6 wells/treatment, 5–7 oocytes /well). One-way ANOVA followed by Tukey’s multiple comparison test or Student–Newman–Keuls (SNK) test was used for statistical analysis. Different letters (a, b, c) denote statistical differences between groups. p < 0.05 was considered statistical significance.

Incubation of zebrafish follicles with PNX-20 significantly increased germinal vesicle breakdown compared to control (Fig. 12). MIH (positive control) showed the highest response compared to both control and PNX-20 treated groups.

## Discussion

PNX is a novel peptide with hormone-like actions to regulate reproduction in mammals. The research outlined here addressed whether PNX-20 has any role in the regulation of reproduction in zebrafish. Our research determined the effects of PNX-20 on all three tissues in the HPG axis, and liver, a major source of vitellogenin that is critical for ovarian follicle biology. Our immunohistochemical studies found PNX-like and SREB3-like immunoreactivity in the gonads, specifically in the supporting cell layers of developing ovarian follicles and spermatocytes. This provides new evidence for testis and ovary as a source of PNX-20 and sites of its action. The local expression suggests a possible role of PNX-20 in zebrafish reproduction. We then used 100 and 1000 ng/g BW PNX-20 to study whether PNX-20 has any role in zebrafish reproduction. A single I.P injection of PNX-20 significantly upregulated the expression of both *sgnrh* and *cgnrh-II* mRNAs in the hypothalamus of zebrafish. Both these GnRHs are expressed in zebrafish, with sGnRH has a pivotal role in the gonadotropin secretion from the pituitary^13^. Results suggest that PNX-20 directly acts on the GnRH system and upregulates the expression of GnRH in both male and female zebrafish. This is consistent with results obtained from mammals^1,3–5^ and non-mammals ^12^. Based on this, it is likely that the stimulatory effect of PNX-20 on the GnRH system is conserved across species. Similarly, we found an upregulation of *kiss1*, *kiss*2 (both male and female fish) and *kiss1rb* (only in males ) mRNA expression in the brain of zebrafish. The kisspeptin system is primarily involved in the regulation of reproduction in vertebrates^14,15^. Increased expression of kisspeptin system in PNX-20 treated fish suggests that in addition to the GnRH, PNX-20 influences another potent regulator of reproduction in the hypothalamus of zebrafish. It was reported that in the rat hypothalamus, PNX stimulates kisspeptin-1 transcription in both arcuate and anteroventral periventricular (AVPV) nucleus^3^. Kiss1 is abundantly expressed in the teleost habenula, a region involved in serotonin mediated behavior responses^16^. Studies in mammals reported that in addition to its reproductive roles, PNX-20 is a potent regulator of behavioral response as well^6,17–19^. Upregulation of kiss 1 post-PNX-20 administration suggests in addition to the reproductive regulatory role, PNX might be involved in the regulation of behavior in teleost. These results present a generally stimulatory role for PNX-20 on the hypothalamic reproductive hormones. Intracerebroventricular injection of PNX- 20 significantly increased the plasma LH levels in a dose-related manner to potentiate GnRH-induced LH secretion from cultured anterior pituitary cells of rats *in vitro*^5^. Due to the extremely small size of zebrafish pituitary gland, we were unable to collect this tissue and determine the effects of PNX-20 on pituitary hormones.

The effects of PNX-20 on zebrafish gonads were also positive. *lhr* mRNA expression in the testis increased, while its expression in the ovary decreased in response to PNX-20. Meanwhile, *fshr* expression was downregulated in the testis and upregulated in the ovary of the treatment group. This differential effect is likely due to the sex-specific variation in the effects of PNX-20. In agreement with the observations in the hypothalamus, an upregulation of *kiss1*, *kiss1rb* and *kiss 2* (only in the male treatment group) mRNAs was identified in the gonads of zebrafish treated with PNX-20. Studies conducted in different fish species suggest that kisspeptin and gonadal development are positively correlated^20^. The upregulation of the kisspeptin system in the gonads of zebrafish is another possible mechanism by which PNX-20 influences zebrafish reproduction. However, we did not observe any significant changes in *kiss1ra* (*kiss1r* or kiss1 receptor a) mRNA expression in the hypothalamus and gonads of zebrafish injected with PNX-20 (data not shown).

Gonadal sex steroids are critical endocrine regulators of reproduction in vertebrates and are essential for the maturation of gametes and the development of secondary sexual characters^21^. Our gene quantification results indicate that PNX-20 upregulates the expression of critical genes involved in gonadal sex steroidogenesis in both male and female zebrafish. This includes the *cyp11a1* (*P450ssc*), *cyp17a1* (17α-hydroxylase) and *cyp19a1a* (gonadal aromatase) in which their expression was significantly increased in both male and female treatment groups. In addition, *17β hsd* mRNA expression significantly increased in the testis of zebrafish injected with PNX-20. Our results suggest that PNX-20 has a role in the sex steroidogenesis in zebrafish. The only exception was the expression of *star*, which was significantly lower in the testis of the 1000 ng/g treatment group in our study. We also measured other reproduction-related genes. One among them is AMH, which has a role in sex determination, gonadal development and gametogenesis in fishes ^22^. Decreased expression of *amh* was observed in the testis of zebrafish treated with PNX-20. AMH is a steroidogenic suppressor in the gonads^22^. Decreased *amh* mRNA expression is additional evidence for the positive role of PNX-20 on sex steroidogenesis in male zebrafish. Another key regulator involved in vertebrate sex determination and gonadal development and gametogenesis is DMRT1^23^. We observed a significant increase in the expression of *dmrt1* mRNA in the testis, but it was decreased in the ovary of the PNX injected fish groups. This stands as another example for the sex-specific influence of PNX-20 on gonadal gene expression. It was reported that DMRT1 mutant zebrafish fail to develop into male fish or become sterile males, but ovary develops normally^24^. In addition, the loss of DMRT1 leads to conditions including gonadal dysgenesis, sex reversal syndromes, and reduced germ cells in the testis of vertebrates^25–28^. Upregulation of potent male reproductive promoter DMRT1 in the testis is additional evidence for the positive influence of PNX-20 in zebrafish gamete biology. Both *ar* and *er* mRNA expression was significantly higher in the ovary, but opposite effects were found in the case of testis. It was reported that in medaka, treatment with estradiol (E2) significantly increases the expression of both ER and AR, but treatment with 11-Ketotestosterone (11-KT) suppresses both AR and ER the brain^29^. It is possible that a similar kind of regulation happens in the gonads of the zebrafish, where the expression of steroidogenic genes and circulating steroid hormones were elevated, but the receptors (AR and ER) were decreased.

In addition to the HPG axis and related hormones, many other factors and biomolecules are important for reproductive success in zebrafish. One among them is vitellogenin, the principal yolk protein, synthesized and secreted predominantly by the liver. Vitellogenin has a critical role in the development and maturation of egg or ova in vertebrates^30^. We used ZFL cells to study the *in vitro* effects of PNX-20 on vitellogenin mRNA expression. We observed that all vitellogenin mRNAs (*vtg 1*, *2*, 3, 4, 5 and 7) were significantly upregulated post-PNX-20 treatment in the ZFL cell line. Our results suggest that the upregulation of the vitellogenin mRNA expression may lead to elevated vitellogenin secretion, which leads to adequate availability of circulating vitellogenin, which then promotes the maturation of egg or developing ova. This is another possible mechanism by which PNX-20 influence (positively) zebrafish reproduction (in female fish). Liver vitellogenin synthesis is under the control of estrogen, which acts via estrogen receptors (esr’s). In our research, we observed that PNX-20 treatment leads to an increased expression of estrogen receptors (*esr-2a* and *esr-2b*), which is an additional (possible) mechanism by which PNX-20 influence vitellogenesis in zebrafish. We also found elevated expression of *shbg*, a liver-derived binding protein involved in the regulation of circulating sex steroid levels in the ZFL cell line post-PNX-20 treatment. In an alternate approach, we used siRNA mediated knockdown to study whether endogenous PNX-20 has any role in vitellogenin synthesis in the ZFL cell line. We found that the siRNA mediated gene knockdown of *smim20* leads to decreased expression of all vitellogenin genes in the ZFL cell line. This suggests endogenous PNX-20 is a regulator of vitellogenin synthesis in zebrafish liver.

To study whether PNX-20 has any role in the maturation of gametes (ova), we used oocyte maturation assay, which is a well-established method to assess the direct action of ligand and hormones on oogenesis/oocyte development^31–34^. The incubation with zebrafish PNX-20 (both 100 and 100 ng/mL PNX-20) upregulated germinal vesicle breakdown and oocyte maturation in zebrafish follicles. This is in addition to the results we observed in both our *in v*ivo and *in vitro* effect of PNX-20 on the overall increase in gene expression. This additional evidence for the direct action of PNX-20 on gamete maturation in zebrafish suggests extra means by which PNX-20 plays a positive modulatory role in fish reproduction.

The outcomes of this research provide several lines of novel information on exogenous PNX-20 in regulating reproduction. These include the regulation of vitellogenin, upregulation of steroidogenic mRNAs in the gonads, and stimulation of oocyte maturation in zebrafish. In addition, we discovered that endogenous PNX is also important for vitellogenin mRNA expression. Our results on the effects of PNX-20 on hypothalamic *gnrh* and kisspeptin mRNAs also concur with what has been reported in mammalian models, and at least in one fish species. These results help to conclude that the regulatory role (generally stimulatory) of PNX on reproduction is conserved across species. However, further studies are needed to confirm that the mRNA expression changes are also reflected at the protein level, both in tissues (enzymes) and in circulation (hormones). Similarly, pituitary expression and secretion of gonadotropins in response to PNX-20 need to be measured. While such limitations exist, this research and its outcomes have provided substantial new information to implicate PNX-20 in fish reproduction. Future studies on its role in other fish species, especially those cultured as a food source is strongly expected to benefit aquaculture.

## Methods

### Animals

Adult male and female zebrafish (*Danio rerio*; 12 months old; body weight: 1 g) were obtained from the Aquatic Toxicology Research Facility at the University of Saskatchewan. Fish were maintained in constantly aerated freshwater aquaria (10 L capacity), under a simulated 12 h light:12 h dark photoperiod (lights on at 07:00 AM). Fish were fed once daily (4% body weight ration) with slow sinking pellets (Aqueon, Franklin, WI, United States) at a scheduled feeding time. Tricaine methanesulfonate-222 (TMS-222, Syndel Laboratories, BC, Canada, 0.5%) was used for anesthetizing the fish (for intraperitoneal [I.P] injection) and euthanasia by spinal transection, followed by tissue collection. All animal studies were carried out according to the policies of the Canadian Council for Animal Care and were approved by the University of Saskatchewan Animal Research Ethics Board (Animal Use Protocol # 2012–0082).

### Immunohistochemial and immunocytochemical localization of PNX and SREB3 in zebrafish gonads and zebrafish liver cell line

Identifying and localizing PNX and SREB3 immunoreactive cells in the gonads will assist us in predicting possible reproductive regulatory roles of PNX in zebrafish. Immunohistochemical studies (IHC) were conducted using methods described earlier^35^. The details of antibodies used in our research are rabbit anti PNX-14 primary antibody (Phoenix Pharmaceuticals, Burlingame, CA, United States, 1:1000 dilution) and rabbit anti-SREB3 antibody - N-terminal (Abcam, Cambridge, UK, 1:500 dilution). The secondary antibody used was Texas Red Goat anti-rabbit antibody (Vector Laboratories, Burlingame, CA, United States, 1:3000 dilution). All antibodies were diluted using antibody diluent (Abcam, UK). Antigen retrieval step was included for slides stained with SREB3 (1 mM EDTA, pH 8.0, and heat treatment, antigen retrieval protocol, Abcam). Preabsorption and secondary antibody alone controls were conducted as previously described^36^. Briefly, for the preabsorption control, zebrafish PNX-20 (custom synthesized, 10 μg) was preabsorbed with PNX-14 primary antibody overnight and this cocktail was used instead of the primary antibody for immunostaining. The negative controls were run only with the secondary antibody (no primary antibody incubation, used antibody diluent). Only the latter was used as a control for the SREB3 immunoreactivity. Vectashield medium containing DAPI dye (Vector Laboratories, Burlingame, CA, United States), which stain the nucleus of the cells blue, was used to mount the slides. The immunocytochemical (ICC) studies were conducted to localize PNX and SREB3 immunoreactivity in the zebrafish liver cell line (ZFL, Cat# ATCC CRL-2643, ATCC, Manassas, VA, United States). The ICC was conducted as previously described^37^ with slight modifications. Briefly, upon 60–70% confluency, cells grown in chamber slides (Corning, VWR, Canada) were fixed using ice-cold methanol (kept overnight at −20) for 2–5 minutes. The cells were then incubated with PBS-PF (5%), followed by cell permeabilization using PBS-Triton X-100 (0.2%) for 10 minutes. The slides were then incubated with blocking solution containing 5% protein block (Abcam) and BSA (5%) in PBS to prevent any non-specific antibody binding. The cells were then incubated with the primary antibody in antibody diluent solution (Abcam, UK) at 4 °C overnight. We used the same antibodies and dilutions as described above in the IHC section. No antigen retrieval steps were used for SREB3 stained slides (paraformaldehyde fixative is not used). Slides were mounted using Vectashield medium containing DAPI (Vector Laboratories). Both IHC and ICC slides were imaged using Olympus BX 51 microscope (Olympus Corporation, Tokyo, Japan) and analyzed using the DP controller software (Olympus Corporation, Japan). Since heterologous antibodies are used here, the immunostaining obtained is referred to as phoenixin-like and SREB3-like, to consider for the possible non-specific binding of antibodies.

### Effect of exogenously administered PNX-20 on reproductive regulatory genes in the hypothalamus and gonads of zebrafish

Male and female zebrafish (age and weight-matched) were grouped (n = 6/group) in fish aquaria and were acclimated for 2 weeks. On the experiment day, fish were anesthetized (TMS-222, 0.5%) and intraperitoneally injected with 0.9% saline (control) or 100 or 1000 ng/g body weight (BW) custom synthesized zebrafish PNX-20 (AGVNQADIQPVGVKVWSDPY, Pacific Immunology, Ramona, CA, United States, Cat# 1711-PAC-21; ≥ 95% pure). The total injection volume used was 4 microliters (μl)/fish. This dose was decided based on our unpublished results from preliminary studies. Tissues (including the hypothalamus, and gonads) were collected 1 hour post-injection and stored at −80 °C until further analysis.

### ZFL cell line maintenance and PNX-20 treatment

The liver is the primary site of synthesis of vitellogenin, the principal component of yolk protein critical for the development and maturation of egg or ova. To study whether PNX-20 has any role in vitellogenin system in zebrafish, we have incubated liver cells with zebrafish PNX-20. Zebrafish liver (ZFL) cell line was purchased from ATCC (ATCC CRL-2643, Manassas, VA, United States) and was cultured in a complete growth media [50 % L-15 (ATCC, VA, United States), 35 % DMEM HG (Gibco, Thermo Fisher Scientific, Sunnyvale, CA, United States), 15 % Ham's F12 (Gibco, Thermo Fisher Scientific, CA, United States), 0.15 g/L sodium bicarbonate (BioShop, Mainway, Burlington, ON, Canada), 15 mM HEPES (Millipore Sigma, Burlington, Massachusetts, United States), 0.01 mg/ml bovine insulin (Sigma-Aldrich, St. Louis, Missouri, United States), 50 ng/ml mouse EGF (Sigma-Aldrich, St. Louis, Missouri, United States), 5% heat-inactivated fetal bovine serum (Gibco, Thermo Fisher Scientific, CA, United States), 0.5% trout serum (Caisson Laboratories, Smithfield, Utah, United States) as suggested by ATCC. The cells were cultured in T25 ml flasks (Greiner Cellstar, VWR, PA, United States) and maintained at 28 degrees in an incubator. Cells were sub-cultured every 3 days, or when they reach 90% confluency. Passages 4–6 were used for all our experiments. For the *in vitro* peptide exposure studies, ZFL cells were cultured in 24 well plates (Greiner Cellstar, VWR, PA, United States) for all our experiments. Briefly, approximately 1 × 10^6^ cells/well were allowed to grow in a 24 well plate for 2 days or until they reach 60–70% confluency. This was followed by replacing the complete growth media with media containing different doses of PNX-20 (0, 1, 10, 100 nM zebrafish PNX-20). All our experiments were run with ‘n’ = 6 wells/treatment, and all experiments were repeated twice. Control wells received complete growth media without any peptides (received an equal amount of sterile water instead) in it. The time points considered are 1, 2, 6, and 24 hr. At each time point, media from cells were removed and 500 μl of RiboZol RNA isolation reagent (aMReSCO, VWR, PA, United States) was added and incubated at room temperature for 5 minutes. This was followed by collecting the samples and stored them at −80 °C for total RNA extraction.

### siRNA mediated knockdown of PNX-20

To study whether endogenous PNX-20 has any effect on the vitellogenin system in zebrafish liver, we used siRNA mediated gene knockdown approach using the ZFL cell line. Zebrafish PNX-20 siRNA and scrambled siRNA were custom synthesized (Dharmacon, Horizon Discovery, Waterbeach, United Kingdom). The scrambled siRNA represents the same composition of nucleotides; however, the arrangement of nucleotides in the sequence is highly dissimilar. Scrambled siRNA is to check or confirm whether the order of nucleotide or nucleotide integrity is critical for siRNA action. Both siRNAs and its scrambled controls were designed using online design tools (Genscript sequence scramble tool). Details of siRNAs are provided in Table 1.

**Table 1:** The table is added with sequence information in siRNA format and correct nomenclature.

Sense sequence	Antisense sequence
UACCUACUUUUCCUGCUUACGCUU	GCGUAAGCAGGAAAAGUAGGUAUU
**Scrambled Sense sequence**	**Scrambled Antisense sequence**
AGUACUUCGCUUCCUUCCUCUUU	AGAGGAAGGAAGCGAAGUACUUU

The ZFL cells grown in 24 well plates (as described above) were treated with either different doses of siRNA (0, 1, 10, 100 nM) or scrambled siRNA. We used Lipofectamine RNAiMAX (Invitrogen, Thermo Fisher scientific CA, United States) mediated siRNA transfection method in our experiment. The Lipofectamine-siRNA mix was prepared in accordance with the manufacturer's instructions. Briefly, Lipofectamine was prepared using Opti-MEM serum-free media (Gibco, Thermo Fisher Scientific, CA, United States) and was mixed and incubated with siRNA, which devoid of any serum. The final concentrations for the treatments were prepared by adding the appropriate volume of the Lipofectamine-siRNA mix to complete growth media containing serum. This mixture was added to cells, which is about 50–60% confluent. The time points we used to check the level of knockdown were 24, 48, 72 and 96 hr post siRNA treatment. The total RNA extraction was carried out as described below. Cell viability and growth rate were closely monitored during the entire treatment period.

### Total RNA extraction, cDNA synthesis, RT-PCR, and real-time quantitative PCR

Total RNA extraction from samples (tissues and cells), cDNA synthesis and RT-qPCR were conducted as described earlier^38^. RiboZol RNA isolation reagent (aMReSCO, VWR, Radnor, PA, United States) was used for total RNA was extraction, followed by DNase 1 (Thermo Fisher Scientific, CA, United States) treatment following the manufactures instructions. The purity and quantity of the total RNA extracted were determined by optical density (OD) absorption ratio (OD 260 nm/OD 280 nm) using a Nanodrop 2000 (Thermo Fisher Scientific, CA, United States). iScript cDNA synthesis kit (BioRad Laboratories, Hercules, CA, United States) was used for cDNA synthesis, following the manufacturer’s instructions. The CFX Connect Real-Time-quantitative PCR Detection System (Bio-Rad, CA, United States) and SensiFAST SYBR No-ROX Kit (Biolne, Froggabio, Toronto, ON, Canada,) were used for gene expression analysis. Livak method^39^ was used for the relative data analysis (2-ΔΔCt method). The quantitative gene expression of different genes and are normalized to the expression of the housekeeping genes, beta-actin and 18 s. Validation and optimization of the primers were carried out prior to qPCR to find the highest efficiency annealing temperatures. The qPCR conditions used were as follows: initial denaturation 95 °C (2 min), 35 cycles of denaturation: 95 °C (5 s), annealing: specific to each gene (25 s), elongation: 72 °C (20 s, when annealing temperature was less than 55 °C). A melting curve analysis was performed at 65 °C to 95 °C (5 s) was run to confirm the absence of any dimer formation or artifacts for each primer set. All samples were run in duplicates. Negative controls with no template DNA (used nuclease-free water instead) in the PCR mix were also run for each gene. The primer sequences and annealing temperatures used for each primer set for quantifying mRNA expression are listed in Table 2. Other than the primers used for vitellogenin genes (except vitellogenin 1), all primers were custom designed. Primer sequences for vitellogenin genes (vtg 2, 3, 4, 5,& 7) were previously reported^40^

**Table 2:** List of primers used for PCR amplification of mRNAs.

Gene	Accession no.	Primer sequence (5′−3′)		Annealing temperature (°C)
		Forward	Reverse	
18 S rRNA	NM_173234.1	GGCGAGGGTTCTGCATAATA	CATCCTTCGTGTCCTCAACA	60
β-actin	NM_131031.2	TTCAAACGAACGACCAACCT	TTCCGCATCCTGAGTCAATG	60
sGnRH	NM_182887.2	CCCGGTGGAAAAAGAAGCGT	CCCCGTCTGTCTGGAAATCTT	60
cGnRH-II	NM_181439.4	AATGCAGTTACCTGAGACCG	AATCACGAATGAGGGCATCC	60
Kiss 1	NM_001113489.1	CTTCTCCATGGGTGCAGGTC	AATCGTGTGAGCATGTCCTGT	60
Kiss 1Rb	NM_001110531.1	CCTTCTGTGCTGAAGACGTG	CTCGGTGCTCCTCCTTTTGA	60
Kiss 2	NM_001142585.1	ATGGAGCGAAGGCAGTTTGA	TGTCAGAGTCGCTGGTTGTC	60
FSH R	NM_001001812.1	TTCCTGCTCAAACCCATTCC	GCATCCAAATCGGCTAGTCA	60
LH R	AY714133.1	TGAAAGAGCAGCCAGGTACT	TGCTAAATTTCCTTTCGCCG	60
CYP11a1	NM_152953.2	ACCCTGCATAAATGAGCGTC	GACAGTGGAGTTTTGCGGTG	60
CYP17a1	NM_212806.3	AGACCCACCACAGACCTTTA	GCACAATCGGCCACTTAAAC	60
CYP19a1a	NM_131154.3	ACCTCCACAAACTCTCACCT	TTGAGCGGGACTCCTAGAAA	60
CYP19a1b	NM_131642.2	TTGGACGCATGCATAAGACA	ACCGAATGGCTGGAAGTAAC	60
StAR	NM_131663.1	CTGTTTTCTGGCTGGGATGT	TCGCATGACAATACAGGTGG	60
17βhsd	NM_205584.2	TGTATCTGGAGGCGATGGAA	TGGAGGTGAGTTTCAGGCTA	60
DMRT1	NM_205628.2	CAGTCGCTCCATGTTGTCTT	AGTGGGCTGGTAAAGGTTGT	60
AMH	NM_001007779.1	TTAAGGGACATCCTGCCTCA	AACACAAATACAGTCGGCGT	60
AR	NM_001083123.1	CTCCAAAGCAAAGGACACCT	TTCGCCCATCTCGTTTTTCA	60
ER	NM_152959.1	GATGTCCCTGCTCACCAACA	CAACACTTCCAGCCAAGAGC	60
PNX-20	NM_001302624.1	TTTGGAGGCTTCGTTGCAG	GGCTTGTAGGGATCAGACCA	57
SREB3	NM_131498.1	CCATTGCCCACCATCGTTTC	ATGAAGCCTAGCGTGTCGTT	60
VTG-1	NM_001044897.3	GAAGACTTTCTCGCCTGGAT	GCAGTACAGCAGTGGTCTAA	60
VTG-2	NM_001044913.1	GGTGACTGGAAGATCCAAG	TCATGCGGCATTGGCTGG	57
VTG-3	NM_131265.1	TTAGAACCAGCAAAGGATGC	CATCTCTTTTCTCCTTAAATAC	57
VTG-4	NM_001045294.2	GAGAGACTGCCAAAAATTGCCT	GGAGAGAAAATCCTTATCAATGGTG	57
VTG-5	NM_001025189.2	CCAAAAATTGTCACCACTTATGCT	CTTCATTCCTCCATGATATGCTTA	60
VTG-7	NM_001102671.1	CCATCTCAGAAGTCCTTTG	CAAACATTCTTGTGAAAATGAAGATA	60
Esr1	NM_152959.1	GACAGAAACCGTCGAAAGAG	CCGCGATCTTTACGAATACC	58
Esr2a	NM_180966.2	GTCCGAGGTCTCAAGAGATA	AGGGCTTCTTCATGTCTTTC	57
Esr2b	NM_174862.3	TCTCAGCACCTCTTTCCT	CATGCTAGCCTCAGTGTATG	60
SHBG	NM_001007151.3	GAGCAGCAGGTGATCAAATA	CAAGGGTTAGGAACTCAA	57

### Oocyte maturation assay

Oocyte maturation assay was conducted as described earlier^31,41^. Briefly, 3–4 female zebrafish were euthanized as described above and ovaries were collected in sorting media containing 90% Leibovitz's L‐15 medium (Sigma, Cat# L1518) at pH 9.0 containing 10% bovine serum albumin (BSA). Individual oocytes or follicles in stage III were carefully separated from others under a dissection microscope. Follicle with size ranges from 0.55–0.62 mm in diameter were chosen for the study (oocytes at this stage respond to the maturation inducing hormone) as previously described^32,33,42^. Collected stage III follicles were either incubated in a 24 well plate with media (90% L-15 media pH 9 + 10% BSA) containing either 100 ng/mL maturation-inducing hormone (MIH; 4-Pregnen-17α,20β-diol-3-one) or 10 or 100 ng/mL ZF PNX-20. The control group was run without any hormones. To each well, 5–7 oocytes were added (n = 6 wells per treatment) and the experiment was repeated 4 times. The maturation rate was scored under a dissection microscope 24 hr post-incubation and the germinal vesicle break down, followed by changes in the appearance of the follicle from opaque to translucent is used to confirm maturation (fifth stage).

### Statistical analysis

Quantitative qPCR gene expression data were analyzed using one-way ANOVA followed by Tukey’s multiple comparison test or Student–Newman–Keuls (SNK) test. p < 0.05 was considered statistical significance. PRISM version 5 (GraphPad Inc., United States) and IBM SPSS version 21 (IBM, United States) software were used for statistical analysis. PRISM version 5 (GraphPad Inc., United States) was used for generating graphs. Data are represented as mean + SEM.

## Data Availability

The data of this study are available from the corresponding author upon reasonable request.
